# Correlative serum biomarker analyses in the phase 2 trial of lenvatinib-plus-everolimus in patients with metastatic renal cell carcinoma

**DOI:** 10.1038/s41416-020-01092-0

**Published:** 2020-10-07

**Authors:** Chung-Han Lee, Robert J. Motzer, Hilary Glen, M. D. Michaelson, James Larkin, Yukinori Minoshima, Michio Kanekiyo, Hiroki Ikezawa, Pallavi Sachdev, Corina E. Dutcus, Yasuhiro Funahashi, Martin H. Voss

**Affiliations:** 1 Department of Medicine, Memorial Sloan Kettering Cancer Center, New York, NY USA; 2 Medical Oncology, Beatson West of Scotland Cancer Center, Glasgow, UK; 3 Massachusetts General Hospital Cancer Center, Boston, MA USA; 4 Department of Medical Oncology, Royal Marsden NHS Foundation Trust, London, UK; 5 Eisai Co., Ltd, Ibaraki, Japan; 6 Eisai Inc., Woodcliff Lake, NJ USA; 7 Eisai, Co., Ltd, Tokyo, Japan

**Keywords:** Tumour biomarkers, Prognostic markers

## Abstract

**Background:**

No biomarkers have been established to predict treatment efficacy in renal cell carcinoma (RCC). In an exploratory retrospective analysis of a Phase 2 study, we constructed composite biomarker scores (CBSs) to predict progression-free survival (PFS) and overall survival (OS) in patients with metastatic RCC randomised to receive lenvatinib-plus-everolimus.

**Methods:**

Of 40 biomarkers tested, the 5 most strongly associated with PFS (HGF, MIG, IL-18BP, IL-18, ANG-2) or OS (TIMP-1, M-CSF, IL-18BP, ANG-2, VEGF) were used to make a 5-factor PFS-CBS or OS-CBS, respectively. A 2-factor CBS was generated with biomarkers common to PFS-CBS and OS-CBS. Patients were divided into groups accordingly (5-factor-CBS high: 3−5, CBS-low: 0–2; 2-factor-CBS high: 1–2, CBS-low: 0).

**Results:**

PFS/OS with lenvatinib-plus-everolimus were significantly longer in the 5-factor CBS-high group versus the CBS-low group (*P* = 0.0022/*P* < 0.0001, respectively). In the CBS-high group, PFS/OS were significantly longer with lenvatinib-plus-everolimus versus everolimus (*P* < 0.001/*P* = 0.0079, respectively); PFS was also significantly longer with lenvatinib-plus-everolimus versus lenvatinib (*P* = 0.0046). The 5-factor-CBS had a predictive role in PFS and OS after multivariate analysis. Similar trends were observed with the 2-factor-CBS for PFS (i.e., lenvatinib-plus-everolimus versus everolimus).

**Conclusions:**

The 5-factor CBS may identify patients with metastatic RCC who would benefit from lenvatinib-plus-everolimus versus everolimus; additional validation is required.

**Clinical trial registration:**

The clinical trial registration number is NCT01136733.

## Background

Several molecularly targeted agents (VEGF receptor and mTOR inhibitors) are approved to treat metastatic renal cell carcinoma (RCC).^1–7^ However, treatment of RCC remains challenging as most patients develop resistance to systemic therapy.^8^ Such difficulties have led to the development of combination therapies (i.e., tyrosine kinase inhibitors with immune checkpoint inhibitors or mTOR inhibitors).^9^ Lenvatinib—an oral multityrosine kinase inhibitor of VEGF receptors 1–3, fibroblast growth factor (FGF) receptors 1–4, platelet-derived growth factor receptor α, RET, and KIT^8,10–12^—in combination with everolimus has demonstrated efficacy in preclinical models of RCC.^13^ In a randomised, 3-arm, Phase 2 study (Study 205) in metastatic RCC, patients assigned to lenvatinib-plus-everolimus combination therapy had significantly improved progression-free survival (PFS) compared with patients who were assigned to everolimus monotherapy (median PFS 14.6 vs 5.5 months; HR 0.40; 95% confidence interval [CI]: 0.24–0.68; *P* < 0.001).^3^ As a result, lenvatinib-plus-everolimus was approved by the US Food and Drug Administration for the treatment of patients with advanced RCC following 1 prior anti-angiogenic therapy.^14,15^

The clinical use of biomarkers (tissue- or blood-based) to predict efficacy of molecularly targeted agents in RCC remains limited.^16^ Intertumoural and intratumoural heterogeneity has been linked with therapeutic failure and the development of drug resistance.^17^ Moreover, the heterogeneity of RCC tumours has made the identification of tissue-based prognostic markers challenging.^18–23^ The use of circulating biomarkers (and composite biomarker scores [CBSs]; derived from peripheral blood proteins), however, remains an active area of investigation in patients with RCC. Importantly, biomarker signatures derived from peripheral blood samples are more easily studied than signatures from tissue; as such, CBSs may serve as much-needed tools for predicting survival in patients with RCC treated by tyrosine-kinase inhibitors^16,24–28^ or mTOR inhibitors.^16^

Biomarker signatures for lenvatinib-plus-everolimus combination therapy are not well studied. We designed CBSs based on protein concentrations in peripheral blood samples of patients with metastatic RCC from Study 205 who were randomised to receive combination therapy. These CBSs were then used to identify patients from Study 205 who were most likely to benefit from lenvatinib-plus-everolimus combination therapy.

## Methods

### Patients

Details on patient eligibility criteria for Study 205 have been published.^3^ Eligible patients were ≥18 years of age with histologically verified clear-cell RCC, had experienced radiographic progression of advanced or metastatic RCC within 9 months of stopping previous VEGF-directed treatment, and had measurable disease per Response Evaluation Criteria In Solid Tumors (RECIST) v1.1.^3^ Patients also had an Eastern Cooperative Oncology Group performance status score of 0 or 1 and adequate organ function.^3^

The source study followed the International Conference on Harmonization Good Clinical Practice guidelines and local regulations and was approved by the respective institutional review board or independent ethics committee at each participating study centre. All patients provided written informed consent before enrolling.

### Study design and treatment

Study 205 was a Phase 2, open-label, multicentre, international study.^3^ Patients were randomly allocated 1:1:1 to receive either lenvatinib (18 mg/day) plus everolimus (5 mg/day), single-agent lenvatinib (24 mg/day), or single-agent everolimus (10 mg/day). Treatment was administered orally once a day in 28-day cycles, and imaging scans were obtained every 8 weeks. Treatment continued until disease progression, unacceptable toxicity, or patient withdrawal of consent occurred.

The primary end point in Study 205 was PFS based on investigator review and RECIST v1.1^3,29^; secondary end points included assessments of safety and tolerability, pharmacokinetic profiles of lenvatinib (as a single-agent and in combination with everolimus), overall survival (OS), and the proportion of patients with an objective response.^3^

### Biomarker analyses

For this retrospective exploratory analysis, 40 candidate biomarkers (Supplementary Table 1) were selected based on prior biomarker analyses conducted in RCC and other diseases and based on a review of the literature.^30–32^ These candidate biomarkers were measured in serum samples, which had been collected at baseline (pre-treatment) and posttreatment (cycle 1 day 15 [C1D15], and cycle 2 day 1 [C2D1]), using 19 preconfigured CustomMAP immunoassay panels. Analytes for which >20% of the samples demonstrated levels below the lower limit of quantification were excluded (Supplementary Table 1). Due to the low sensitivity of CustomMAP immunoassay for FGF-23 (57% of samples showed levels below the lower limit of quantification), and the importance of this biomarker for the FGF receptor pathway, levels of FGF-23 were quantified using a conventional ELISA assay (Kainos Laboratories, Inc., Bunkyo-ku, Tokyo, Japan) and included in target engagement analyses for FGF-receptor inhibition.

### Construction of the 5-factor CBS for PFS and OS

Associations of baseline levels of each biomarker with PFS in patients treated with lenvatinib-plus-everolimus were individually assessed using a univariate Cox regression model with either continuous values or dichotomised populations with median cut-off followed by a log-rank test with or without a false discovery rate (FDR) adjustment. In a subsequent step, the 5 serum biomarkers with the strongest individual associations on univariate testing with median cut-off values (positive or negative, by HR) for PFS were used to construct the 5-factor PFS-CBS (following a framework for constructing CBSs similar to that developed by Voss et al.^16^; Supplementary Fig. 1). To calculate patients’ 5-factor PFS-CBSs, each biomarker was designated a score based on its level at baseline (with the median level of each biomarker as the cut-off). As such, findings for each biomarker were integrated into a CBS by assigning a value of 1 (biomarker level fell within the range associated with a longer PFS in the single biomarker univariate analysis) or 0 (biomarker fell within the range associated with a shorter PFS in the single biomarker univariate analysis) for each of the 5; the sum of the individual values determined the 5-factor PFS-CBS for each patient.

The construction of the 5-factor OS-CBS was similar to the construction of the 5-factor PFS-CBS, with 1 modification: the 5 serum biomarkers with the strongest individual associations (positive or negative, by HR) for OS on univariate analysis with median cut-off values were used to construct the 5-factor OS-CBS (i.e., values of 1 for biomarker levels within the range associated with a longer OS based on univariate testing or 0 if levels were associated with a shorter OS) (Supplementary Fig. 1).

Associations between total CBS scores and clinical outcomes with therapy were then investigated in separate analyses for PFS and OS, applying the PFS-CBS and OS-CBS, respectively. Based on the respective CBS value, patients were divided into two groups: low and high. A low score was defined as 0–2 and a high score was defined as 3–5. These cut-offs were selected based on the largest or smallest HR from a survival analysis (PFS) in the lenvatinib-plus-everolimus arm, with significant differences by Cox regression analysis and with significance by multivariate analysis between CBS, treatment arms, and PFS.

### Construction of the 2-factor CBSs

To determine if a simpler, less labour-intensive CBS was predictive of positive outcomes, we generated a 2-factor CBS, using the 2 serum biomarkers that were common to both the 5-factor PFS-CBS and 5-factor OS-CBS (Supplementary Fig. 1). Computation of patients’ individual scores followed the same approach as outlined above (notably, biomarker associations with univariate analyses—using median cut-off—were similar for PFS and OS). In the 2-factor CBS, a low score was defined as 0 and a high score was 1–2. Cut-offs were selected based on the approach used for the 5-factor CBSs detailed above.

### Statistical methods

Statistical analyses included all patients in the biomarker analysis set—defined as a subset of the full analysis set (intention-to-treat population) with at least 1 biomarker measurement; analyses were performed using SAS v9.3 or higher (SAS Institute Inc.). Given the exploratory nature of these analyses *P*-values should be considered nominal.

### Serum pharmacodynamic biomarker analysis

Changes in serum biomarker concentrations were measured from baseline (cycle 1 day 1 [C1D1]) at C1D15 and C2D1 and were summarised for each treatment arm using a 1-sample Wilcoxon signed-rank test. In addition, a 2-sample Wilcoxon rank-sum test was conducted to compare the changes in biomarker levels between the lenvatinib-plus-everolimus arm and either the lenvatinib monotherapy or everolimus monotherapy arms.

### Single baseline serum biomarker analyses

For single biomarker analyses, the HRs of PFS and OS for biomarkers with continuous values were first calculated between measured values with 1 standard deviation (HR per standard deviation). For biomarkers that had a significant association with PFS and OS in the lenvatinib-plus-everolimus arm, the cut-off analysis (with median value) was conducted by univariate Cox regression and log-rank tests. The differences in associations between biomarker levels (higher than median and lower than median) and PFS and OS among treatment arms were assessed using multivariate Cox regression with treatment arms, biomarker level, and their interaction.

### Comparison of survival outcomes and objective response rate (ORR) between CBS groups in individual treatment arms

Associations between CBS groups (high vs low) and either PFS or OS were analysed using univariate Cox regression and log-rank tests in each treatment arm (i.e., lenvatinib monotherapy, everolimus monotherapy, and lenvatinib-plus-everolimus). Multivariate Cox regression analyses were performed to examine the associations between CBS group and PFS or OS adjusted by risk group (International Metastatic RCC Database Consortium [IMDC] risk group: favourable vs intermediate/poor), within the lenvatinib-plus-everolimus arm. Analyses for PFS and OS were performed for the 5-factor and 2-factor CBS groups. Associations between 5-factor CBS groups and ORR were assessed based on Fisher’s exact test for each treatment arm.

### Comparison of survival outcomes between treatment arms in each CBS group

Associations between treatment groups (lenvatinib-plus-everolimus vs lenvatinib; and lenvatinib-plus-everolimus vs everolimus) and PFS and OS were analysed using univariate Cox regression and log-rank tests in each CBS group (high and low). Associations between treatment arms and PFS and OS in each CBS group (high and low) were also assessed using multivariate Cox regression with treatment arms, CBS groups, and their interaction with a Kaplan–Meier curve. Analyses were performed for both the 5-factor and 2-factor CBS groups.

## Results

### Patients

Of the 153 patients included in the intent-to-treat population, 96.1% (total *n* = 147 patients; lenvatinib-plus-everolimus, *n* = 49; lenvatinib, *n* = 51; everolimus, *n* = 47) had serum samples taken for the biomarker analysis. Patient demographics and baseline characteristics were generally similar across treatment arms (Table 1). Most patients were deemed to have IMDC intermediate or poor risk (lenvatinib-plus-everolimus: 85.4%; lenvatinib: 86.3%; everolimus: 80.9%). Additional details on patient disposition have been reported.^3^

**Table 1:** Patient demographics and characteristics.

Parameter	Serum biomarker analysis set			Full analysis set (*N* = 153)
	LEN + EVE (*n* = 49)	LEN (*n* = 51)	EVE (*n* = 47)	
Median age, years (range)	61.0 (44, 79)	64.0 (41, 79)	58.0 (37, 77)	61.0 (37, 79)
Males, *n* (%)	33 (67.3)	38 (74.5)	35 (74.5)	112 (73.2)
ECOG PS, *n* (%)				
0	26 (53.1)	28 (54.9)	27 (57.4)	84 (54.9)
1	23 (46.9)	23 (45.1)	20 (42.6)	69 (45.1)
MSKCC risk group, *n* (%)^a^				
Favourable	11 (22.4)	11 (21.6)	12 (25.5)	35 (22.9)
Intermediate	19 (38.8)	17 (33.3)	18 (38.3)	56 (36.6)
Poor	19 (38.8)	23 (45.1)	17 (36.2)	62 (40.5)
IMDC risk group, *n* (%)				
Favourable	7 (14.6)	7 (13.7)	9 (19.1)	24 (15.8)
Intermediate	31 (64.6)	32 (62.7)	27 (57.4)	94 (61.8)
Poor	10 (20.8)	12 (23.5)	11 (23.4)	34 (22.4)
Median duration of most recent prior VEGF-targeted therapy, months, months (range)	9.6 (2.0, 66.2)	13.5 (0.7, 81.8)	8.8 (1.6, 57.8)	11.5 (0.7, 81.8)

Percentages are based on the number of patients with nonmissing values.

*ECOG PS* Eastern Cooperative Oncology Group performance status, *EVE* everolimus, *IMDC* International Metastatic renal cell carcinoma Database Consortium, *LEN* lenvatinib, *MSKCC* Memorial Sloan Kettering Cancer Center, *VEGF* vascular endothelial growth factor.

^a^The 3-point MSKCC score was used for this analysis.^49^

### Serum pharmacodynamic biomarker analysis

Pharmacodynamic biomarkers previously associated with other VEGFR-tyrosine kinase inhibitors (i.e., VEGF, VEGF-D, ANG-2, TIE-2, VEGFR-2, and VEGFR-3) significantly changed in all three treatment arms (at C1D15), as assessed by a 1-sample Wilcoxon signed-rank test (Supplementary Fig. 2A). Among these biomarkers, TIE-2, VEGFR-2 and VEGFR-3, had significantly greater decreases with lenvatinib-plus-everolimus combination therapy compared with either monotherapy (by a 2-sample Wilcoxon rank-sum test) (Supplementary Fig. 2B).

### Association of baseline serum biomarkers with improved survival in patients treated with lenvatinib-plus-everolimus

A single biomarker (IL-18BP) was significantly associated with PFS by univariate Cox regression analysis with continuous values after FDR adjustments (HR: 1.720 [95% CI: 1.226, 2.413]; adjusted *P* = 0.0457) (Supplementary Table 2); however, a dichotomised analysis based on median cut-off values suggested that IL-18BP was not associated with PFS (*P* = 0.1508).

For identification of biomarkers associated with OS, 12 candidate baseline serum biomarkers were identified by univariate Cox regression analysis with continuous values after FDR adjustments. Of these biomarkers, only 10 were associated with OS via a dichotomised analysis based on median cut-off values. Only 5 of these 10 biomarkers (at low baseline concentrations; FGF-21, ICAM-1, IL-18BP, M-CSF and VEGFR-3) were identified as having the potential to be predictive of a longer OS in the lenvatinib-plus-everolimus arm compared with either the lenvatinib or everolimus monotherapy arms using multivariate Cox regression with treatment arms, biomarker level, and their interaction (Supplementary Table 3).

### Survival analyses according to 5-factor CBS groups

To explore if multi-serum biomarker signatures could provide stronger predictive signals for survival than individual biomarkers, 5-factor CBSs were constructed using the 5 candidate markers with the strongest associations (positive or negative, by HR) for PFS (i.e., 5-factor PFS-CBS) and OS (i.e., 5-factor OS-CBS), respectively, on univariate analysis. The 5-factor PFS-CBS was comprised of HGF, MIG, IL-18BP, IL-18, and ANG-2 (Table 2), and the 5-factor OS-CBS included TIMP-1, M-CSF, IL-18BP, ANG-2 and VEGF (Table 2).

**Table 2:** Biomarkers associated with PFS and OS in the lenvatinib-plus-everolimus arm, as determined by HR.

Marker	Cut-off		Low group		High group		Log-rank *P* value		Direction of HR: high/low
	Quantile	Value	*n*	MST	*n*	MST		With FDR	HR (95% CI)
Association with PFS									
ANG-2	0.5	6.800 μg/L	27	20.1	21	5.9	0.0351	0.1508	2.360 (1.040–5.355)
HGF	0.5	7.350 μg/L	28	20.1	20	5.6	0.0264	0.1508	2.550 (1.088–5.974)
IL-18	0.5	264.0 ng/L	28	14.7	20	5.6	0.0317	0.1508	2.418 (1.058–5.526)
IL-18BP	0.5	17.0 μg/L	24	17.5	24	6.9	0.0305	0.1508	2.431 (1.059–5.580)
M-CSF	0.5	0.9650 μg/L	28	14.7	20	7.4	0.1144	0.3088	1.904 (0.845–4.288)
MIG	0.5	1250 ng/L	30	20.1	18	7.4	0.0299	0.1508	2.506 (1.062–5.914)
TIMP-1	0.5	199.0 μg/L	26	14.7	22	5.6	0.0392	0.1508	2.326 (1.023–5.291)
VEGF	0.5	305.0 ng/L	29	14.7	19	11.2	0.2526	0.5247	1.597 (0.711–3.587)
Association with OS									
ANG-2	0.5	6.800 μg/L	27	NE	21	21.7	0.003	0.0137	3.005 (1.402–6.442)
HGF	0.5	7.350 μg/L	28	32.2	20	20.5	0.0081	0.0258	2.592 (1.249–5.377)
IL-18	0.5	264.0 ng/L	28	32.1	20	20.7	0.0163	0.0367	2.376 (1.149–4.915)
IL-18BP	0.5	17.00 μg/L	24	32.2	24	18.4	0.0008	0.0073	3.469 (1.605–7.501)
M-CSF	0.5	0.9650 μg/L	28	NE	20	14.5	0.0002	0.0044	3.765 (1.781–7.959)
MIG	0.5	1250 ng/L	30	25.5	18	25.5	0.9668	0.9668	0.985 (0.472–2.056)
TIMP-1	0.5	199.0 μg/L	26	NE	22	16.1	0.0003	0.0044	3.770 (1.741–8.162)
VEGF	0.5	305.0 ng/L	29	32.2	19	20.5	0.0021	0.0137	2.993 (1.441–6.213)

Biomarkers most strongly associated with PFS or OS (by HR), respectively, are shaded grey.

*ANG-2* angiopoietin-2, *FDR* false discovery rate, *HGF* hepatocyte growth factor, *HR* hazard ratio, *IL-18* interleukin-18, *IL-18BP* interleukin-18 binding protein, *M-CSF* macrophage colony-stimulating factor, *MIG* monokine induced by gamma interferon, *MST* median survival time, *NE* not estimable, *OS* overall survival, *PFS* progression-free survival, *TIMP-1* tissue inhibitor of metalloproteinase-1, *VEGF* vascular endothelial growth factor.

### Patients in the 5-factor PFS-CBS-high group benefitted from lenvatinib-plus-everolimus treatment

**Fig. 1:**
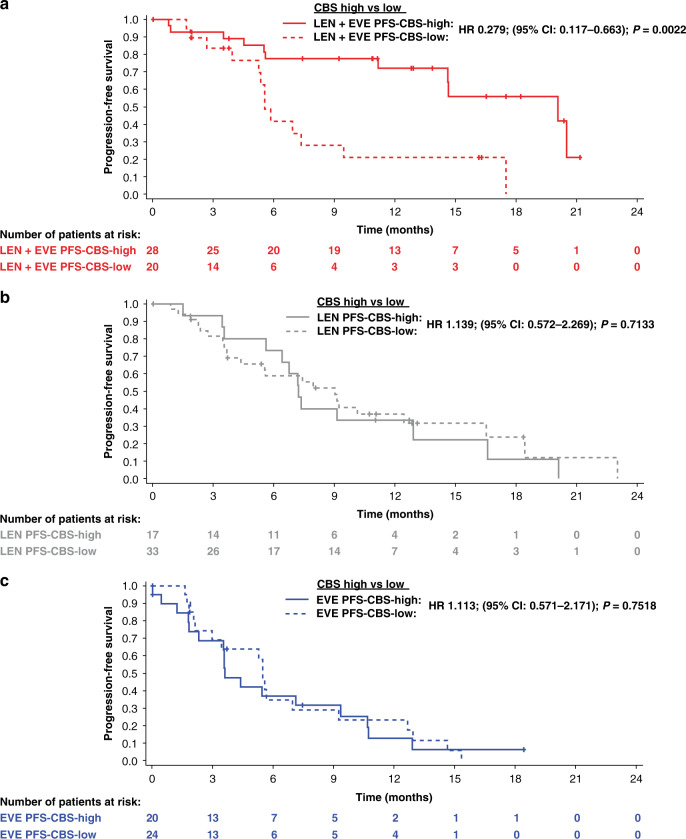
Kaplan–Meier curves of PFS for PFS-CBS (5-factor)-high groups compared to PFS-CBS-low groups within treatment arms. **a** lenvatinib + everolimus; **b** lenvatinib and **c** everolimus.

In the lenvatinib-plus-everolimus treatment arm, PFS was significantly longer in the CBS-high group (median: 20.1 months) compared with the CBS-low group (median: 5.6 months; HR 0.279; 95% CI: 0.117–0.663; *P* = 0.0022) (Fig. 1 and Supplementary Table 4). An association between PFS and CBS group in the lenvatinib-plus-everolimus treatment arm was supported by a multivariate Cox regression model adjusted by IMDC risk group (favourable vs intermediate/poor; HR 0.285; 95% CI 0.119–0.679) (Supplementary Table 4). Conversely, a significant difference in PFS was not observed between the CBS-high and CBS-low groups in patients randomly assigned to lenvatinib or everolimus monotherapy (Fig. 1; Supplementary Table 4).

In the CBS-high group, PFS was significantly longer with lenvatinib-plus-everolimus (median: 20.1 months) compared with lenvatinib (median: 7.2 months; HR 0.317; 95% CI: 0.138–0.731; *P* = 0.0046) or everolimus (median: 3.6 months; HR 0.186; 95% CI 0.080–0.429; *P* < 0.001) (Supplementary Table 4). However, in the CBS-low group, there was no significant difference in PFS with lenvatinib-plus-everolimus versus lenvatinib or everolimus treatment (Supplementary Table 4). Multivariate Cox regression analysis further indicated that the CBS-high group was predictive of longer PFS with lenvatinib-plus-everolimus versus lenvatinib (*P*_interaction_ = 0.0098) or everolimus (*P*_interaction_ = 0.0154) treatment (Supplementary Table 4).

### Patients in the 5-factor OS-CBS-high group benefitted from lenvatinib-plus-everolimus treatment

**Fig. 2:**
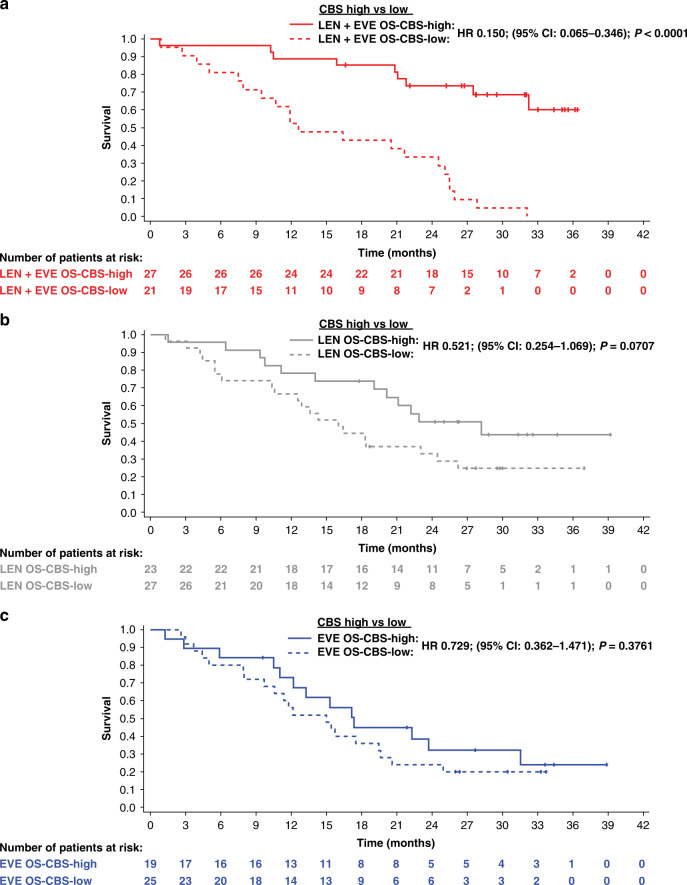
Kaplan–Meier curves of OS for OS-CBS (5-factor)-high groups compared with OS-CBS-low groups within treatment arms. **a** lenvatinib + everolimus; **b** lenvatinib and **c** everolimus.

OS was significantly longer in the CBS-high group (median was not reached) compared with the CBS-low group (median: 12.6 months; HR 0.150; 95% CI 0.065–0.346; *P* < 0.0001) in the lenvatinib-plus-everolimus treatment arm (Fig. 2 and Supplementary Table 4). The association was maintained when adjusting for IMDC risk group (favourable vs intermediate/poor) by multivariate Cox regression analysis (HR 0.165; 95% CI 0.068–0.401) (Supplementary Table 4). In contrast, among patients randomised to receive either lenvatinib or everolimus monotherapy, no significant difference in OS was observed when stratified by OS-CBS score (Fig. 2 and Supplementary Table 4).

In the CBS-high group, OS was significantly longer with lenvatinib-plus-everolimus (median was not reached) compared with everolimus (median: 17.4 months; HR 0.331; 95% CI 0.141–0.779; *P* = 0.0079), but not compared with lenvatinib (median: 28.2 months; HR 0.518; 95% CI 0.217–1.234; *P* = 0.1307) (Supplementary Table 4). No significant differences in OS were observed between treatment arms in the CBS-low group. Overall, multivariate Cox regression analysis indicated that the CBS-high group was predictive of a longer OS with lenvatinib-plus-everolimus versus everolimus (*P*_interaction_ = 0.0125).

### ORR analyses according to PFS-CBS and OS-CBS (5-factor) groups

**Fig. 3:**
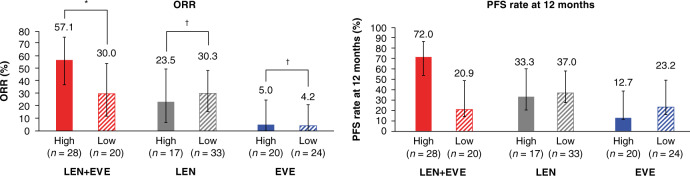
ORR and 12 month landmark PFS rate is stratified by PFS-CBS-high groups and PFS-CBS-low groups (5 factor). ORR and PFS rate in PFS-CBS-high (5 factor) groups and PFS-CBS-low groups.

In the lenvatinib-plus-everolimus arm, ORR was significantly higher in the OS-CBS-high group versus the OS-CBS-low group (63.0% vs 23.8%, respectively; Fisher’s exact test *P* < 0.01) (Supplementary Fig. 3); there was a trend towards a higher ORR in the PFS-CBS-high group versus the PFS-CBS-low group (Fig. 3). The respective ORRs did not vary significantly between OS-CBS high versus low groups or PFS-CBS high versus low groups for lenvatinib or everolimus (Supplementary Fig. 3 and Fig. 3). Similar trends were observed for the PFS rate at 12 months (Fig. 3).

### Analyses according to CBS (2-factor) groups

We constructed the 2-factor CBS using the common factors identified in the 5-factor OS- and PFS-CBSs (i.e., ANG-2 and IL-18BP) to explore if a simpler, less labour-intensive, biomarker signature (compared with 5-factor CBS) could predict survival.

### Survival was improved with lenvatinib-plus-everolimus versus lenvatinib for patients in the 2-factor CBS-high group

**Fig. 4:**
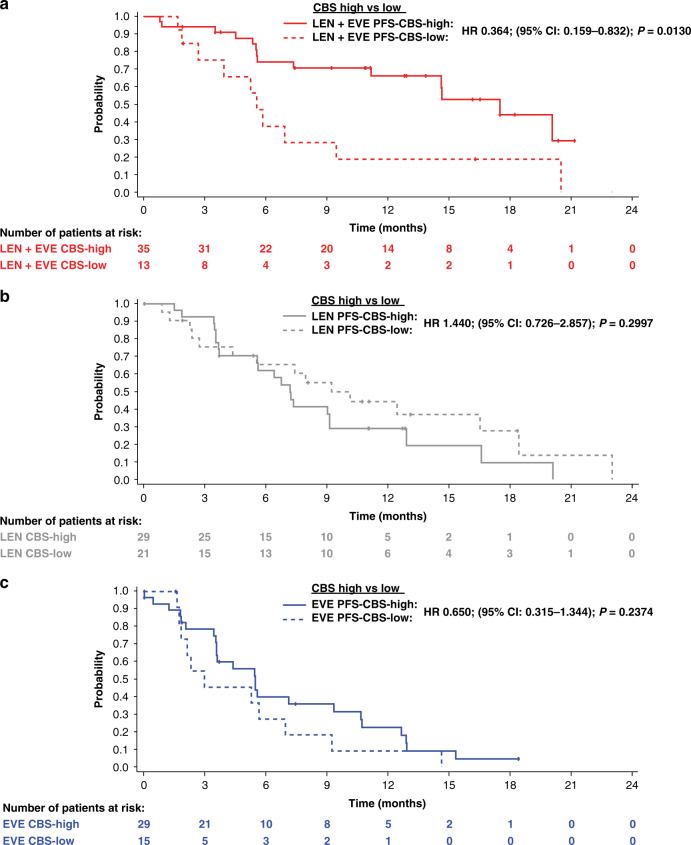
Kaplan–Meier curves of PFS for CBS 2-factor CBS-high groups compared with CBS-low groups within treatment arms. **a** lenvatinib + everolimus, **b** lenvatinib and **c** everolimus.

In the lenvatinib-plus-everolimus treatment arm PFS was significantly longer in the CBS-high group (median: 17.5 months) compared with the CBS-low group (median: 5.6 months; HR 0.364; 95% CI 0.159–0.832; *P* = 0.0130) (Fig. 4 and Supplementary Table 5); additionally OS was significantly longer in the CBS-high group (median: 32.1 months) compared with the CBS-low group (median 11.9 months; HR 0.213; 95% CI 0.098–0.459; *P* < 0.0001) (Supplementary Fig. 4 and Supplementary Table 5). Multivariate analysis adjusted by IMDC risk group (favourable vs intermediate/poor) suggested a nonsignificant association (or trend) between PFS and CBS group (HR 0.444; 95% CI 0.189–1.043) in the lenvatinib-plus-everolimus arm and a preserved significant association between OS and CBS group (HR 0.249; 95% CI 0.113–0.548) (Supplementary Table 5).

In the CBS-low group, PFS and OS did not vary significantly between the 3 treatment arms (i.e., lenvatinib-plus everolimus, lenvatinib, or everolimus) (Supplementary Table 5). However, in the CBS-high group, PFS was significantly longer with lenvatinib-plus-everolimus than with lenvatinib (HR 0.358; 95% CI 0.179–0.716; *P* = 0.0026) or everolimus (HR 0.254; 95% CI 0.128–0.506; *P* < 0.0001) (Supplementary Table 5). Moreover, in the CBS-high group, OS was significantly longer with lenvatinib-plus-everolimus than with everolimus (HR 0.504; 95% CI 0.263–0.967; *P* = 0.0359) (Supplementary Table 5). Multivariate Cox regression analyses indicated that the CBS-high group was predictive of a longer PFS and OS with lenvatinib-plus-everolimus versus lenvatinib (*P*_interaction_ = 0.0070 and *P*_interaction_ = 0.0377, respectively) but not versus everolimus (*P*_interaction_ = 0.2297 and *P*_interaction_ = 0.2125, respectively) (Supplementary Table 5).

## Discussion

We constructed CBS models according to levels of circulating biomarkers in the blood serum of patients with metastatic RCC from Study 205 at baseline. These models were then used to identify subgroups of patients who might have an enhanced response to lenvatinib-plus-everolimus treatment. Additionally, we confirmed that known molecular targets of lenvatinib (e.g., VEGF receptors 1–3, FGF receptors 1–4)^8,33^ and everolimus (e.g., mTOR C1)^34^ are modulated by treatment.

Target engagement of lenvatinib monotherapy with VEGF receptors and FGF receptors was indicated by increases in serum levels of VEGF and FGF-23 from baseline. Moreover, target engagement of everolimus monotherapy with immune or inflammatory response pathways was indicated by decreases from baseline in serum levels of various proinflammatory cytokines and chemokines, including IL-18, ITAC/CXCL11, IP-10/CXCL10 (significant at C1D15 only), MCP-1, and RANTES (Supplementary Fig. 2A). Interestingly, consistent significant decreases (at both C1D15 and C2D1) of ITAC/CXCL11 and IP-10/CXCL10 were not detected with lenvatinib-plus-everolimus combination therapy. On the other hand, large decreases in serum levels of TIE-2, VEGF receptor-2, and VEGF receptor-3 (all of which have been reported as pharmacodynamic biomarkers for VEGFR-tyrosine kinase inhibitors^35^) provided evidence of target engagement of lenvatinib-plus-everolimus combination therapy and were suggestive of enhanced anti-angiogenesis activity compared with each monotherapy (Supplementary Fig. 2B).

Although no single biomarker has been verified as a prognostic indicator in metastatic RCC,^15^ other groups have developed CBSs to predict efficacy.^24,25,27,28^ Specifically, in a Phase 2 study of first-line sunitinib versus first-line everolimus for patients with metastatic RCC, everolimus was found to be inferior to sunitinib as a first-line treatment (as measured by PFS).^36^ A subsequent analysis, however, showed that baseline levels of numerous circulating biomarkers correlated with a survival benefit for everolimus and/or sunitinib treatment.^16^ Correspondingly, analyses showed that PFS was similar for first-line everolimus and first-line sunitinib in patients with CBS-high,^16^ thereby suggesting that everolimus may be an effective first-line treatment in a specific subpopulation.

In our analysis, biomarkers with the strongest association to PFS (HGF, MIG, IL-18BP, IL-18, and ANG-2) and OS (TIMP-1, M-CSF, IL-18BP, ANG-2 and VEGF) were included in separate PFS-CBS and OS-CBS, respectively. The association of these biomarkers with angiogenesis (HGF, ANG-2, VEGF, TIMP-1)^37–41^ and immune and inflammatory responses (MIG, IL-18BP, IL-18, and M-CSF)^42–44^ suggests that these 2 signalling pathways play an important role in successful treatment with lenvatinib-plus-everolimus.^16,45^

In Study 205, PFS was significantly longer for lenvatinib-plus-everolimus compared with everolimus monotherapy (median 14.6 vs 5.5 months; HR 0.40, 95% CI 0.24–0.68; *P* = 0.0005).^3^ OS, however, was only numerically longer with lenvatinib-plus-everolimus combination therapy (median 25.5 vs 17.5 months; HR 0.55, 95% CI 0.30–1.01; *P* = 0.062). Our analysis identified specific populations of patients (defined by their CBS) in Study 205 that were most likely to benefit from lenvatinib-plus-everolimus combination therapy. Specifically, patients in the PFS-CBS-high (5-factor) and OS-CBS-high (5-factor) group appeared to have improved PFS and OS with lenvatinib-plus-everolimus combination therapy compared with everolimus monotherapy. Although the simpler, less labour-intensive, 2-factor CBS appeared more robust than a single serum biomarker analysis and showed promise as a prognostic tool for PFS, it did not appear to predict OS.

It should be noted that, while some roles of the biomarkers used in both the 2-factor PFS- and OS-CBS are known (ANG-2 is thought to facilitate angiogenesis and IL-18BP suppresses IL-18),^42,46^ further analysis of the role of ANG-2 and IL-18BP in the signalling pathways of metastatic RCC should be further investigated if 2-factor CBSs are to be used to classify patients with metastatic RCC for treatment.

This exploratory analysis was limited by the number of patients in each CBS group and its retrospective nature. Moreover, biomarker levels were dichotomised according to their median concentration in this study, making the results more challenging to translate to the clinic. As such, the results will require independent validation at set biomarker cut-offs. Additional biomarker studies may also be considered—it could be of clinical utility to determine why PFS and OS are associated with different biomarker signatures and to analyse the serum biomarker signature at various timepoints throughout patients’ treatments (to clarify how peripheral measurements correlate with changes in the tumour).

CBS can be a powerful tool because it can be assessed based on a single blood draw obtained at baseline without the need for a tumour biopsy. From a clinical perspective, the reduced invasiveness of such a tool is advantageous because of a reduction in medical complications and lower healthcare costs.^47^ The results from our analysis suggest that patients with metastatic RCC and categorised as CBS-high may benefit from second-line treatment with lenvatinib-plus-everolimus. Conversely, patients with low CBS score may not derive added benefit from combination therapy over monotherapy. This distinction (if validated) is clinically relevant because of the notable differences in toxicity between lenvatinib-plus-everolimus combination therapy and everolimus monotherapy–including rates of grade 3 or 4 adverse events, dose reductions, and treatment discontinuation for adverse events.^3^ Additional studies to determine the utility of the CBS as a prognostic tool for patients with metastatic RCC are warranted.

## Supplementary information

Supplemental Material

## Data Availability

The data will not be available for sharing at this time as the data are commercially confidential. However, Eisai will consider written requests to share the data on a case-by-case basis.
