# Supplementary material for: Correlative serum biomarker analyses in the phase 2 trial of lenvatinib-plus-everolimus in patients with metastatic renal cell carcinoma
Source: Br J Cancer. 2020 Oct 7;124(1):237–46. doi: 10.1038/s41416-020-01092-0 (PMC7782770; doi:10.1038/s41416-020-01092-0)
Supplement: Supplementary file 1 — Supplemental Material [file 41416_2020_1092_MOESM1_ESM.docx]

**SUPPLEMENTAL TABLES**

**Supplemental Table 1.** Summary of LLOQ for serum assay of candidate biomarkers

| **Analyte** | **Under LLOQ (%)** | **Analyte** | **Under LLOQ (%)** |
| --- | --- | --- | --- |
| ANG-1 | 0 | ITAC | 15 |
| ANG-2 | 0 | KIM-1 | 9.7 |
| CRP | 0.2 | M-CSF | 1.5 |
| EOTAXIN1 | 8.1 | MCP-1 | 8.7 |
| FGF-21 | 1 | MIG | 0.7 |
| FGF-23 | 57 | MIP-1α | 85.6 |
| G-CSF | 44.6 | MMP-9 | 97.8 |
| HGF | 0 | PLGF | 11.1 |
| ICAM-1 | 0 | RANTES | 0 |
| IFN | 100 | SDF-1 | 0 |
| IL-10 | 91.3 | TIE-2 | 0.7 |
| IL-12P70 | 100 | TIMP-1 | 0 |
| IL-13 | 96.8 | TNF- α | 92.3 |
| IL-18 | 3.2 | TNFR2 | 0 |
| IL-18BP | 0 | VCAM-1 | 0 |
| IL-1β | 98 | VEGF | 1.2 |
| IL-4 | 97.3 | VEGF-D | 2.5 |
| IL-6 | 49 | VEGFR-1 | 99.5 |
| IL-8 | 18.8 | VEGFR-2 | 0 |
| IP-10 | 0.2 | VEGFR-3 | 2.2 |

Analytes for which >20% of the samples demonstrated levels below the lower limit of quantification
are highlighted in gray.

ANG-1, angiopoietin 1; ANG-2, angiopoietin 2; CRP, C-reactive protein; EOTAXIN1, also known as CCL11 or C-C motif chemokine ligand 11; FGF-21, fibroblast growth factor 21; FGF-23, fibroblast growth factor 23; G-CSF, granulocyte-colony stimulating factor; HGF, hepatocyte growth factor; ICAM-1, intercellular adhesion molecule 1; IFN, interferon; IL-10, interleukin 10; IL-12P70, interleukin 12, p70; IL-13, interleukin 13; IL-18, interleukin 18; IL-18BP, interleukin 18 binding protein; IL-1β, interleukin 1 beta; IL-4, interleukin 4; IL-6, interleukin 6; IL-8, interleukin 8; IP-10, interferon gamma-induced protein 10; ITAC, interferon-inducible T-cell alpha [chemoattractant](https://en.wikipedia.org/wiki/Chemoattractant); KIM-1, kidney injury molecule 1; LLOQ, lower limit of quantification; M-CSF, macrophage-colony stimulating factor; MCP-1, monocyte chemoattractant protein-1; MIG, monokine induced by gamma interferon; MIP-1α, major intrinsic protein of lens fiber α; MMP-9, matrix metallopeptidase 9; PLGF, placental growth factor; RANTES, also known as CCLF, chemokine C-C motif ligand 5; SDF-1, stromal cell-derived factor 1; TIE-2, angiopoietin-1 receptor; TIMP1, tissue inhibitor of metalloproteinase-1; TNF-α, tumor necrosis factor α, TNFR2, tumor necrosis factor receptor 2; VCAM-1, vascular cell adhesion protein 1; VEGF, vascular endothelial growth factor; VEGF-D, vascular endothelial growth factor D; VEGFR-1, vascular endothelial growth factor receptor 1; VEGFR-2, vascular endothelial growth factor receptor 2; VEGFR-3, vascular endothelial growth factor receptor 3.

**Supplemental Table 2.** Associations of baseline biomarker levels with PFS in the lenvatinib-plus-everolimus arm with univariate Cox analyses

| **Marker** | **LEN+EVE** | | | | **LEN** | | | | **EVE** | | | |
| --- | --- | --- | --- | --- | --- | --- | --- | --- | --- | --- | --- | --- |
|  | **n** | **HR**  **(95% CI)** | ***P* Value** | ***P* Value with FDR** | **n** | **HR**  **(95% CI)** | ***P* Value** | ***P* Value with FDR** | **n** | **HR**  **(95% CI)** | ***P* Value** | ***P* Value with FDR** |
| ANG-1 | 48 | 0.943  (0.650–1.368) | 0.7579 | 0.7871 | 50 | 0.980  (0.663–1.449) | 0.9188 | 0.9565 | 46 | 1.000  (0.737–1.358) | 0.9978 | 0.9978 |
| ANG-2 | 48 | 26.572  (2.641–267.362) | 0.0054 | 0.0724 | 50 | 1.551  (0.057–42.358) | 0.7949 | 0.8943 | 44 | 1.004  (0.850–1.185) | 0.9637 | 0.9978 |
| CRP | 48 | 1.047  (0.784–1.400) | 0.7541 | 0.7871 | 50 | 1.143  (0.803–1.626) | 0.4592 | 0.6991 | 44 | 1.270  (0.887–1.818) | 0.1921 | 0.8858 |
| EOTAXIN1 | 48 | 0.859  (0.600–1.228) | 0.4042 | 0.5457 | 50 | 0.797  (0.537–1.184) | 0.2619 | 0.6991 | 45 | 1.022  (0.724–1.443) | 0.9020 | 0.9978 |
| FGF-21 | 48 | 1.259  (0.943–1.680) | 0.1183 | 0.3195 | 50 | 3.732  (1.904–7.316) | 0.0001 | 0.0034 | 46 | 0.979  (0.543–1.763) | 0.9424 | 0.9978 |
| HGF | 48 | 2.954  (1.202–7.259) | 0.0182 | 0.1637 | 50 | 1.652  (0.601–4.542) | 0.3303 | 0.6991 | 46 | 1.109  (0.910–1.353) | 0.3062 | 0.8858 |
| ICAM-1 | 48 | 1.322  (0.876–1.993) | 0.1832 | 0.3658 | 50 | 0.894  (0.660–1.209) | 0.4660 | 0.6991 | 45 | 0.975  (0.685–1.386) | 0.8860 | 0.9978 |
| IL-18 | 48 | 1.274  (0.887–1.829) | 0.1897 | 0.3658 | 50 | 0.950  (0.658–1.372) | 0.7829 | 0.8943 | 45 | 1.154  (0.716–1.859) | 0.5561 | 0.9978 |
| IL-18BP | 48 | 1.720  (1.226–2.413) | 0.0017 | 0.0457 | 50 | 0.913  (0.637–1.308) | 0.6195 | 0.8013 | 46 | 1.207  (0.837–1.740) | 0.3149 | 0.8858 |
| IL-8 | 48 | <0.001  (<0.001–>9999) | 0.5138 | 0.6305 | 50 | 2.303  (<0.001–>9999) | 0.9565 | 0.9565 | 45 | 0.972  (0.823–1.148) | 0.7377 | 0.9978 |
| IP-10 | 48 | 1.345  (0.764–2.367) | 0.3043 | 0.4624 | 50 | 0.553  (0.330–0.925) | 0.0240 | 0.3240 | 45 | 1.205  (0.798–1.819) | 0.3749 | 0.8858 |
| ITAC | 48 | 1.251  (0.858–1.824) | 0.2439 | 0.4116 | 50 | 0.985  (0.691–1.404) | 0.9328 | 0.9565 | 46 | 1.273  (0.852–1.902) | 0.2379 | 0.8858 |
| KIM-1 | 48 | 1.538  (0.911–2.595) | 0.1071 | 0.3195 | 50 | 1.240  (0.990–1.551) | 0.0607 | 0.5465 | 45 | 2.378  (1.455–3.885) | 0.0005 | 0.0148 |
| M-CSF | 48 | 1.369  (0.907–2.065) | 0.1345 | 0.3302 | 50 | 1.187  (0.825–1.707) | 0.3555 | 0.6991 | 46 | 1.036  (0.737–1.458) | 0.8382 | 0.9978 |
| MCP-1 | 48 | 1.247  (0.792–1.965) | 0.3405 | 0.4839 | 50 | 0.863  (0.549–1.356) | 0.5225 | 0.7424 | 45 | 1.038  (0.813–1.326) | 0.7632 | 0.9978 |
| MIG | 48 | 1.788  (1.041–3.071) | 0.0353 | 0.2336 | 50 | 0.693  (0.410–1.169) | 0.1693 | 0.6991 | 45 | 1.264  (0.965–1.655) | 0.0888 | 0.8858 |
| PLGF | 48 | 0.942  (0.018–48.529) | 0.9765 | 0.9765 | 50 | 10.453  (0.308–355.255) | 0.1920 | 0.6991 | 46 | 1.101  (0.928–1.306) | 0.2692 | 0.8858 |
| RANTES | 48 | 0.926  (0.628–1.366) | 0.6994 | 0.7869 | 50 | 1.135  (0.814–1.584) | 0.4542 | 0.6991 | 46 | 0.970  (0.660–1.426) | 0.8768 | 0.9978 |
| SDF-1 | 48 | 1.336  (0.834–2.142) | 0.2285 | 0.4113 | 50 | 0.848  (0.558–1.289) | 0.4403 | 0.6991 | 46 | 1.172  (0.843–1.630) | 0.3442 | 0.8858 |
| TIE-2 | 48 | 1.715  (1.012–2.906) | 0.0451 | 0.2336 | 50 | 1.140  (0.831–1.562) | 0.4168 | 0.6991 | 46 | 1.093  (0.771–1.550) | 0.6159 | 0.9978 |
| TIMP-1 | 48 | 1.138  (0.888–1.457) | 0.3083 | 0.4624 | 50 | 1.405  (0.958–2.061) | 0.0818 | 0.5518 | 46 | 1.203  (0.777–1.861) | 0.4074 | 0.8858 |
| TNFR2 | 48 | 1.463  (0.997–2.146) | 0.0519 | 0.2336 | 50 | 1.208  (0.784–1.861) | 0.3910 | 0.6991 | 46 | 1.289  (0.917, 1.811) | 0.1438 | 0.8858 |
| VCAM-1 | 48 | 1.250  (0.897–1.741) | 0.1871 | 0.3658 | 50 | 0.852  (0.554–1.310) | 0.4654 | 0.6991 | 46 | 1.147  (0.806–1.632) | 0.4462 | 0.8858 |
| VEGF | 48 | 1.800  (0.923–3.513) | 0.0846 | 0.3195 | 50 | 1.204  (0.735–1.972) | 0.4620 | 0.6991 | 45 | 1.136  (0.920–1.402) | 0.2369 | 0.8858 |
| VEGF-D | 48 | 1.120  (0.732–1.713) | 0.6026 | 0.7074 | 50 | 1.051  (0.764–1.445) | 0.7620 | 0.8943 | 45 | 0.977  (0.687–1.389) | 0.8954 | 0.9978 |
| VEGFR-2 | 48 | 0.875  (0.588–1.303) | 0.5122 | 0.6305 | 50 | 0.749  (0.519–1.082) | 0.1231 | 0.6646 | 46 | 0.872  (0.607–1.253) | 0.4593 | 0.8858 |
| VEGFR-3 | 48 | 1.501  (0.919–2.451) | 0.1047 | 0.3195 | 50 | 1.078  (0.799–1.455) | 0.6232 | 0.8013 | 46 | 0.917  (0.600–1.402) | 0.6889 | 0.9978 |

ANG-1, angiopoietin 1; ANG-2, angiopoietin 2; CI, confidence interval; CRP, C-reactive protein; EOTAXIN1, also known as CCL11 or C-C motif chemokine ligand 11; EVE, everolimus; FDR, false discovery rate; FGF-21, fibroblast growth factor 21; HGF, hepatocyte growth factor; HR, hazard ratio; ICAM-1, intercellular adhesion molecule 1; IL-18, interleukin 18; IL-18BP, interleukin 18 binding protein; IL-8, interleukin 8; IP-10, interferon gamma-induced protein 10; ITAC, interferon-inducible T-cell alpha [chemoattractant](https://en.wikipedia.org/wiki/Chemoattractant); KIM-1, kidney injury molecule 1; LEN, lenvatinib; M-CSF, macrophage-colony stimulating factor; MCP-1, Monocyte chemoattractant protein-1; MIG, monokine induced by gamma interferon; PLGF, placental growth factor; PFS, progression-free survival; RANTES, also known as CCLF, chemokine C-C motif ligand 5; SDF-1, stromal cell-derived factor 1; TIE-2, angiopoietin-1 receptor; TIMP1, tissue inhibitor of metalloproteinase-1; VCAM-1, vascular cell adhesion protein 1; VEGF, vascular endothelial growth factor; VEGF-D, vascular endothelial growth factor D; VEGFR-2, vascular endothelial growth factor receptor 2; VEGFR-3, vascular endothelial growth factor receptor 3.

**Supplemental Table 3.** Association of baseline biomarker levels with OS in the lenvatinib-plus-everolimus arm with dichotomized analyses using median cutoff points and multivariate Cox analyses

| **Marker** | | | ANG-2 | FGF-21 | HGF | ICAM-1 | IL-18BP | M-CSF | PLGF | TIMP-1 | TNFR2 | VCAM-1 | VEGF | VEGFR-3 |
| --- | --- | --- | --- | --- | --- | --- | --- | --- | --- | --- | --- | --- | --- | --- |
| **Cutoff** | | | 0.5 | 0.5 | 0.5 | 0.5 | 0.5 | 0.5 | 0.5 | 0.5 | 0.5 | 0.5 | 0.5 | 0.5 |
| **Value** | | | 6.800  μg/L | 0.3200  μg/L | 7.350  μg/L | 151.0  μg/L | 17.00  μg/L | 0.9650 μg/L | 55.00 ng/L | 199.0 μg/L | 13.00 μg/L | 754.0 μg/L | 305.0 ng/L | 42.00 μg/L |
| **LEN/EVE** | Low group | n | 27 | 25 | 28 | 27 | 24 | 28 | 29 | 26 | 27 | 26 | 29 | 25 |
|  |  | MST (Mo) | NE | 32.2 | 32.2 | 32.2 | 32.2 | NE | 27.8 | NE | 32.1 | 25.9 | 32.2 | 32.1 |
|  | High group | n | 21 | 23 | 20 | 21 | 24 | 20 | 19 | 22 | 21 | 22 | 19 | 23 |
|  |  | MST (Mo) | 21.7 | 20.5 | 20.5 | 12.6 | 18.4 | 14.5 | 21.7 | 16.1 | 16.4 | 23.6 | 20.5 | 12.6 |
|  | Log-rank | *P* value | 0.003 | 0.0142 | 0.0081 | 0.0028 | 0.0008 | 0.0002 | 0.1113 | 0.0003 | 0.0065 | 0.4920 | 0.0021 | 0.0484 |
|  |  | with FDR | 0.0137 | 0.0349 | 0.0258 | 0.0137 | 0.0073 | 0.0044 | 0.2003 | 0.0044 | 0.0252 | 0.6992 | 0.0137 | 0.1005 |
|  | HR (95% CI) | | 3.005 (1.402–6.442) | 2.474 (1.174–5.214) | 2.592 (1.249–5.377) | 2.916 (1.398–6.081) | 3.469 (1.605–7.501) | 3.765 (1.781–7.959) | 1.786 (0.866–3.683) | 3.770 (1.741–8.162) | 2.674 (1.284–5.573) | 1.287 (0.629–2.635) | 2.993 (1.441–6.213) | 2.052 (0.992– 4.242) |
| **LEN** | Low group | n | 20 | 19 | 23 | 23 | 21 | 24 | 21 | 22 | 25 | 22 | 21 | 20 |
|  |  | MST (Mo) | 25.5 | 22.2 | 24.4 | 21.1 | 22.2 | 22.9 | 28.2 | NE | 22.9 | 20.1 | 26.2 | 26.2 |
|  | High group | n | 30 | 31 | 27 | 27 | 29 | 26 | 29 | 28 | 25 | 28 | 29 | 30 |
|  |  | MST (Mo) | 17.4 | 19.1 | 18.3 | 18.4 | 16.4 | 16.2 | 16.0 | 14.2 | 16.0 | 19.7 | 19.1 | 15.1 |
|  | Log-rank | *P* value | 0.1613 | 0.5181 | 0.5939 | 0.8538 | 0.9218 | 0.2687 | 0.0163 | 0.0036 | 0.1694 | 0.6291 | 0.3686 | 0.1009 |
|  |  | with FDR | 0.4573 | 0.7771 | 0.8271 | 0.8866 | 0.9218 | 0.6003 | 0.1466 | 0.0481 | 0.4573 | 0.8271 | 0.6635 | 0.4023 |
|  | HR (95% CI) | | 1.680 (0.807–3.495) | 1.269 (0.611–2.636) | 1.211 (0.601–2.437) | 0.936 (0.466–1.881) | 1.035 (0.514–2.087) | 1.483 (0.736–2.989) | 2.467 (1.155–5.273) | 2.968 (1.383–6.368) | 1.626 (0.807–3.275) | 1.192 (0.587–2.418) | 1.397 (0.673–2.899) | 1.834 (0.879–3.826) |
| Interaction with LEN+EVE | | *P* value | 0.3222 | 0.2523 | 0.1642 | 0.0225 | 0.0288 | 0.1138 | 0.4763 | 0.7490 | 0.3516 | 0.8358 | 0.1787 | 0.8062 |
| **EVE** | Low group | n | 23 | 25 | 21 | 21 | 19 | 20 | 20 | 23 | 19 | 24 | 21 | 24 |
|  |  | MST (Mo) | 22.3 | 15.3 | 19.5 | 13.3 | 23.7 | 17.2 | 19.5 | 19.5 | 19.6 | 17.5 | 17.2 | 13.3 |
|  | High group | n | 21 | 21 | 25 | 24 | 27 | 26 | 26 | 23 | 27 | 22 | 24 | 22 |
|  |  | MST (Mo) | 11.8 | 19.5 | 15.3 | 18.5 | 12.2 | 15.4 | 12.2 | 15.0 | 15.0 | 12.0 | 15.0 | 16.6 |
|  | Log-rank | *P* value | 0.0085 | 0.3885 | 0.2494 | 0.2330 | 0.0866 | 0.6968 | 0.0637 | 0.2050 | 0.4005 | 0.5797 | 0.4060 | 0.1100 |
|  |  | with FDR | 0.2284 | 0.5481 | 0.4809 | 0.4809 | 0.3597 | 0.7526 | 0.3597 | 0.4742 | 0.5481 | 0.7358 | 0.5481 | 0.3713 |
|  | HR (95% CI) | | 2.538  (1.241–5.190) | 0.741 (0.373–1.469) | 1.492 (0.752–2.961) | 0.660 (0.332–1.312) | 1.845 (0.906–3.758) | 1.146 (0.577– 2.274) | 1.919 (0.953–3.867) | 1.548 (0.783–3.061) | 1.347 (0.671–2.705) | 1.210 (0.616–2.376) | 1.339 (0.671– 2.674) | 0.575 (0.289–1.143) |
| Interaction with LEN+EVE | | *P* value | 0.9292 | 0.0223 | 0.3481 | 0.0039 | 0.2999 | 0.0362 | 0.7560 | 0.1299 | 0.2350 | 0.8409 | 0.1602 | 0.0070 |

ANG-2, angiopoietin 2; CI, confidence interval; EVE, everolimus; FDR, false discovery rate; FGF-21, fibroblast growth factor 21; HGF, hepatocyte growth factor; HR, hazard ratio; ICAM-1, intercellular adhesion molecule 1; IL-18BP, interleukin 18 binding protein; LEN, lenvatinib; M-CSF, macrophage-colony stimulating factor; Mo, month; MST, mean square for treatment; NE, not estimable; NT, not tested; OS, overall survival; PLGF, placental growth factor; TIMP-1, tissue inhibitor of metalloproteinase-1; TNFR2, tumor necrosis factor receptor 2; VCAM-1, vascular cell adhesion protein 1; VEGF, vascular endothelial growth factor; VEGFR-3, vascular endothelial growth factor receptor 3.

**Supplemental Table 4.** 5-Factor PFS- and OS-CBS analyses

| **PFS**  **HR between CBS groups in each treatment arm** | | | | | | | | | | | | | | | | | | | |
| --- | --- | --- | --- | --- | --- | --- | --- | --- | --- | --- | --- | --- | --- | --- | --- | --- | --- | --- | --- |
| **Treatment arm** | | | **CBS-high** | | | | | | **CBS-low** | | | | | | | | **HR (95% CI)** | | ***P* value** |
|  |  |  | **n** | | **Median PFS (months)** | | | | **n** | | **Median PFS (months)** | | | | | |  |  |  |
| LEN + EVE | | | 28 | | 20.1 | | | | 20 | | 5.6 | | | | | | 0.279 (0.117–0.663) | | 0.0022 |
| LEN | | | 17 | | 7.2 | | | | 33 | | 9.0 | | | | | | 1.139 (0.572–2.269) | | 0.7133 |
| EVE | | | 20 | | 3.6 | | | | 24 | | 5.5 | | | | | | 1.113 (0.571–2.171) | | 0.7518 |
| **PFS**  **HR between treatment arms in each CBS group** | | | | | | | | | | | | | | | | | | | |
| **CBS** | | **LEN + EVE vs LEN** | | | | | | | | | | | | **LEN + EVE vs EVE** | | | | | |
|  |  | **HR (95% CI); *P*-value** | | | | | | **Interaction *P* value** | | | | | | **HR (95% CI); *P* value** | | | **Interaction *P* value** | | |
| High | | 0.317 (0.138–0.731); 0.0046 | | | | | | 0.0098 | | | | | | 0.186 (0.080–0.429); <0.001 | | | 0.0154 | | |
| Low | | 1.423 (0.708–2.860); 0.3261 | | | | | |  |  |  |  |  |  | 0.646 (0.310–1.348); 0.2387 | | |  |  |  |
| **OS**  **HR between CBS groups in each treatment arm** | | | | | | | | | | | | | | | | | | | |
| **Treatment arm** | | | | **CBS-high** | | | | | | **CBS-low** | | | | | **HR (95% CI)** | | | ***P* value** | |
|  |  |  |  | **n** | | **Median OS (months)** | | | | **n** | | **Median OS (months)** | | |  |  |  |  |  |
| LEN + EVE | | | | 27 | | NE | | | | 21 | | 12.6 | | | 0.150 (0.065–0.346) | | | <0.0001 | |
| LEN | | | | 23 | | 28.2 | | | | 27 | | 16.0 | | | 0.521 (0.254–1.069) | | | 0.0707 | |
| EVE | | | | 19 | | 17.4 | | | | 25 | | 15.0 | | | 0.729 (0.362–1.471) | | | 0.3761 | |
| **OS**  **HR between treatment arms in each CBS group** | | | | | | | | | | | | | | | | | | | |
| **CBS** | **LEN + EVE vs LEN** | | | | | | | | | | | | **LEN + EVE vs EVE** | | | | | | |
|  | **HR (95% CI); *P*-value** | | | | | | **Interaction *P* value** | | | | | | **HR (95% CI); *P*-value** | | | **Interaction *P* value** | | | |
| High | 0.518 (0.217–1.234); 0.1307 | | | | | | 0.0609 | | | | | | 0.331 (0.141–0.779); 0.0079 | | | 0.0125 | | | |
| Low | 1.457 (0.787–2.698); 0.2284 | | | | | |  |  |  |  |  |  | 1.205 (0.651–2.228); 0.5560 | | |  |  |  |  |

Multivariate analysis between PFS and PFS-CBS group adjusted by IMDC risk group (favorable versus intermediate/poor) in the lenvatinib-plus-everolimus arm had a HR 0.285; 95% CI 0.119–0.679.

Multivariate analysis between OS and OS-CBS group adjusted by IMDC risk group (favorable versus intermediate/poor) in the lenvatinib-plus-everolimus arm had a HR 0.165; 95% CI 0.068–0.401.

CBS, composite biomarker score; CI, confidence interval; EVE, everolimus; HR, hazard ratio; IMDC, International Metastatic RCC Database Consortium; LEN, lenvatinib; OS, overall survival; PFS, progression-free survival; RCC, renal cell carcinoma.

| **PFS**  **HR between CBS groups in each treatment arm** | | | | | | | | | | |
| --- | --- | --- | --- | --- | --- | --- | --- | --- | --- | --- |
| **Treatment arm** | | **CBS-high** | | | **CBS-low** | | | **HR (95% CI)** | | ***P* value** |
|  |  | **n** | **Median PFS (months)** | | **n** | **Median PFS (months)** | |  |  |  |
| LEN + EVE | | 35 | 17.5 | | 13 | 5.6 | | 0.364 (0.159–0.832) | | 0.0130 |
| LEN | | 29 | 7.2 | | 21 | 9.2 | | 1.440 (0.726–2.857) | | 0.2997 |
| EVE | | 29 | 5.5 | | 15 | 3.0 | | 0.650 (0.315–1.344) | | 0.2374 |
| **PFS**  **HR between treatment arms in each CBS group** | | | | | | | | | | |
| **CBS** | **LEN + EVE vs LEN** | | | | | | **LEN + EVE vs EVE** | | | |
|  | **HR (95% CI); *P*-value** | | | **Interaction  *P* value** | | | **HR (95% CI); *P*-value** | | **Interaction  *P* value** | |
| High | 0.358 (0.179–0.716); 0.0026 | | | 0.0070 | | | 0.254 (0.128–0.506); <0.0001 | | 0.2297 | |
| Low | 1.634 (0.716–3.729); 0.2402 | | |  |  |  | 0.557 (0.238–1.399); 0.2178 | |  |  |
| **OS**  **HR between CBS groups in each treatment arm** | | | | | | | | | | |
| **Treatment arm** | | **CBS-high** | | | **CBS-low** | | | **HR (95% CI)** | | ***P* value** |
|  |  | **n** | **Median OS (months)** | | **n** | **Median OS (months)** | |  |  |  |
| LEN + EVE | | 35 | 32.1 | | 13 | 11.9 | | 0.213 (0.098–0.459) | | <0.0001 |
| LEN | | 29 | 22.9 | | 21 | 16.0 | | 0.665 (0.331–1.336) | | 0.2486 |
| EVE | | 29 | 17.5 | | 15 | 11.4 | | 0.461 (0.226–0.941) | | 0.0294 |
| **OS**  **HR between treatment arms in each CBS group** | | | | | | | | | | |
| **CBS** | **LEN + EVE vs LEN** | | | | | | **LEN + EVE vs EVE** | | | |
|  | **HR (95% CI); *P* value** | | | **Interaction *P* value** | | | **HR (95% CI); *P* value** | | **Interaction *P* value** | |
| High | 0.626 (0.317–1.234); 0.1725 | | | 0.0377 | | | 0.504 (0.263–0.967); 0.0359 | | 0.2125 | |
| Low | 1.885 (0.890–3.994); 0.0928 | | |  |  |  | 0.949 (0.439–2.054); 0.8945 | |  |  |

**Supplemental Table 5.** 2-factor PFS- and OS-CBS analyses

Multivariate analysis between PFS and CBS group adjusted by IMDC risk group (favorable versus intermediate/poor) in the lenvatinib-plus-everolimus arm had a HR 0.444; 95% CI 0.189–1.043.

Multivariate analysis between OS and OS-CBS group adjusted by IMDC risk group (favorable versus intermediate/poor) in the lenvatinib plus everolimus arm had a HR 0.249; 95% CI 0.113–0.548.

CBS, composite biomarker score; CI, confidence interval; EVE, everolimus; HR, hazard ratio; IMDC, International Metastatic RCC Database; LEN, lenvatinib; NE, not estimable; OS, overall survival; PFS, progression-free survival; RCC, renal cell carcinoma.

**Supplemental Table 6.** Institutional review boards and independent ethics committees by country and site.

| **Country** | **Site** | **IRB/IEC** |
| --- | --- | --- |
| Czech  Republic | 2001 | *(Central EC)*  Eticka komise Fakultni nemocnice u sv. Anny v Brne  Pekarska 53  656 91, Brno  *(Local EC)*  Eticka komise  Masarykuv onkologicky ustav  Zluty kopec 543/7  656 53, Brno |
| Czech  Republic | 2002 | *(Central EC)*  Eticka komise Fakultni nemocnice u sv. Anny v Brne  Pekarska 53  656 91, Brno  *(Local EC)*  Eticka komise Nemocnice Na Bulovce  Budinova 2  180 01, Praha 8 |
| Czech  Republic | 2003 | *(Central EC)*  Eticka komise Fakultni nemocnice u sv. Anny v Brne  Pekarska 53  656 91, Brno  *(No Local EC used)* |
| Czech  Republic | 2004 | *(Central EC)*  Eticka komise Fakultni nemocnice u sv. Anny v Brne  Pekarska 53  656 91, Brno  *(Local EC)*  Eticka komise  Fakultni nemocnice v Motole  V Uvalu 84  150 06, Praha 5 |
| Czech  Republic | 2005 | *(Central EC)*  Eticka komise Fakultni nemocnice u sv. Anny v Brne  Pekarska 53  656 91, Brno  *(Local EC)*  Eticka komise Fakultni nemocnice Olomouc  I.P. Pavlova 6  779 00, Olomouc |
| Poland | 4001 | *(Central EC)*  Niezalezna Komisja Bioetyczna ds. Badan Naukowych przy Gdanskim Uniwersytecie Medycznym  ul. Marii Sklodowskiej-Curie 3A  80-210 Gdansk  *(No Local EC used)* |
| Poland | 4002 | *(Central EC)*  Niezalezna Komisja Bioetyczna ds. Badan Naukowych przy Gdanskim Uniwersytecie Medycznym  ul. M. Sklodowskiej Curie 3A  80-210 Gdansk  *(No Local EC used)* |
| Poland | 4003 | *(Central EC)*  Niezalezna Komisja Bioetyczna ds. Badan Naukowych przy Gdanskim Uniwersytecie Medycznym  ul. Marii Sklodowskiej-Curie 3A  80-210 Gdansk  *(No Local EC used)* |
| Poland | 4004 | *(Central EC)*  Niezalezna Komisja Bioetyczna ds. Badan Naukowych przy Gdanskim Uniwersytecie Medycznym  ul. Marii Sklodowskiej-Curie 3A  80-210 Gdansk  *(No Local EC used)* |
| Spain | 3001 | *(Central EC)*  Agencia de Ensayos Clinicos  Escalera 6B, Sotano, Hospital Clinic  C/Villarroel, 170  Barcelona 08036  *(No Local EC used)* |
| Spain | 3002 | *(Central EC)*  Agencia de Ensayos Clinicos  Hospital Clinic, Escalera 6B, Sotano,  C/Villarroel, 170  Barcelona 08036  *(No Local EC used)* |
| Spain | 3004 | *(Central EC)*  Agencia de Ensayos Clinicos  Escalera 6B, Sotano, Hospital Clinic  C/Villarroel, 170  Barcelona 08036  *(No Local EC used)* |
| Spain | 3005 | *(Central EC)*  Agencia de Ensayos Clinicos  Escalera 6B, Sotano, Hospital Clinic  C/Villarroel, 170  Barcelona 08036  *(No Local EC used)* |
| United  Kingdom | 7001 | *(Central EC)*  London-Surrey Borders REC  South London REC Office (1)  St. George’s University of London  Cranmer Terrace  London, SW17 0RE  *(No Local EC used)* |
| United  Kingdom | 7003 | *(Central EC)*  NRES Committee London – Surrey Borders  Research Ethics Committee (REC) London Centre  Ground Floor  Skipton House  80 London Road  London, SE1 6LH  *(Previous Information)*  London-Surrey Borders REC  South London REC Office (1)  St. George’s University of London  Cranmer Terrace  London, SW17 0RE  *(No Local EC used)* |
| United  Kingdom | 7005 | *(Central EC)*  NRES Committee London – Surrey Borders  Charing Cross Hospital  Research Ethics Committee (REC) Centre  Charing Cross  Room 12, 4^th^ Floor West  Fulham Palace Road  London, W6 8RF  *(Previous Information)*  London-Surrey Borders REC  South London REC Office (1)  St. George’s University of London  Cranmer Terrace  London, SW17 0RE  *(No Local EC used)* |
| United  Kingdom | 7006 | *(Central EC)*  NRES Committee London – Surrey Borders  Charing Cross Hospital  Research Ethics Committee (REC) Centre  Charing Cross  Room 12, 4^th^ Floor West  Fulham Palace Road  London, W6 8RF  *(Previous Information)*  London-Surrey Borders REC  South London REC Office (1)  St. George’s University of London  Cranmer Terrace  London, SW17 0RE  *(No Local EC used)* |
| United  Kingdom | 7007 | *(Central EC)*  London-Surrey Borders REC  South London REC Office (1)  St. George’s University of London  Cranmer Terrace  London, SW17 0RE  *(No Local EC used)* |
| United  Kingdom | 7009 | *(Central EC)*  NRES Committee London – Surrey Borders  Research Ethics Committee (REC) London Centre  Ground Floor  Skipton House  80 London Road  London, SE1 6LH  *(Previous Information)*  London-Surrey Borders REC  South London REC Office (1)  St. George’s University of London  Cranmer Terrace  London, SW17 0RE  *(No Local EC used)* |
| United  Kingdom | 7010 | *(Central EC)*  NRES Committee London – Surrey Borders  Research Ethics Committee (REC) London Centre  Ground Floor  Skipton House  80 London Road  London, SE1 6LH  *(Previous Information)*  London-Surrey Borders REC  South London REC Office (1)  St. George’s University of London  Corridor 1, Room 1.13  1^st^ Floor, Jenner Win, Tooting  London, SW17 0RE  *(No Local EC used)* |
| United  Kingdom | 7011 | *(Central EC)*  NRES Committee London – Surrey Borders  Charing Cross Hospital  Research Ethics Committee (REC) Centre  Charing Cross  Room 12, 4^th^ Floor West  Fulham Palace Road  London, W6 8RF  *(Previous Information)*  London-Surrey Borders REC  South London REC Office (1)  St. George’s University of London  Cranmer Terrace  London, SW17 0RE  *(No Local EC used)* |
| United  Kingdom | 7012 | *(Central EC)*  NRES Committee London – Surrey Borders  Research Ethics Committee (REC) London Centre  Ground Floor  Skipton House  80 London Road  London, SE1 6LH  *(No Local EC used)* |
| United  Kingdom | 7014 | *(Central EC)*  NRES Committee London – Surrey Borders  Research Ethics Committee (REC) London Centre  Ground Floor  Skipton House  80 London Road  London, SE1 6LH  *(No Local EC used)* |
| United  Kingdom | 7015 | *(Central EC)*  NRES Committee London – Surrey Borders  Research Ethics Committee (REC) London Centre  Ground Floor  Skipton House  80 London Road  London, SE1 6LH  *(No Local EC used)* |
| United States | 1001 | Memorial Sloan Kettering Cancer Center Institutional Review Board  1275 York Ave  New York, NY 10065 |
| United States | 1002 | Office for Human Research Studies  Dana Farber Cancer Institute  450 Brookline Avenue, OS229  Boston, MA 02215 |
| United States | 1003 | Western Institutional Review Board  South Hill Business & Technology Center  1019 39^th^ Avenue S.E.  Puyallup, WA 98374 |
| United States | 1006 | Quorum Review IRB  1501 Forth Avenue, Suite 800  Seattle, WA 98101  *(Previous Address)*  1601 Fifth Avenue, Suite 1000  Seattle, WA 98101 |
| United States | 1013 | Office for the Protection of Research Subjects  11000 Kinross Avenue, Suite 211  Los Angeles, CA 90095  *(Previous Information)*  UCLA Office of the Human Research Protection Program  11000 Kinross Avenue, Suite 102  Los Angeles, CA 90095-1694 |
| United States | 1015 | North Mississippi Health Services IRB  830 South Gloster Street  Tupelo, MS 38801 |
| United States | 1016 | Medical University of South Carolina Office of Research Integrity (ORI) Institutional Review Board for Human Research  Harborview Office Tower  19 Hagood Avenue, Suite 601  Charleston, SC 29425 |
| United States | 1017 | Sharp Healthcare Institutional Review Board  8695 Spectrum Center Boulevard  San Diego, CA 92123 |
| United States | 1019 | Quorum Review IRB  1601 Fifth Avenue, Suite 1000  Seattle, WA 98101 |
| United States | 1022 | AAMC Institutional Review Board  2001 Medical Parkway  Annapolis, MD 21401 |
| United States | 1023 | Western Institutional Review Board  1019 39^th^ Avenue S.E.  Suite 120  Puyallup, WA 98374  *(Previous Address)*  3535 Seventh Avenue, SW  Olympia, WA 98502-5010 |
| United States | 1024 | Carolinas Healthcare System IRB  1000 Blythe Boulevard  P.O. Box 32861  Charlotte, NC 28203 |
| United States | 1028 | Quorum Review IRB  1601 Fifth Avenue, Suite 1000  Seattle, WA 98101 |
| United States | 1031 | University of Kansas Medical Center Human Subjects Committee  3901 Rainbow Boulevard  Kansas City, KS 66160 |

EC, ethics committee; IEC, independent ethics committee; IRB, institutional review board.

**SUPPLEMENTAL FIGURES**

**Supplemental Figure 1.** Development of 5-factor and 2-factor CBS


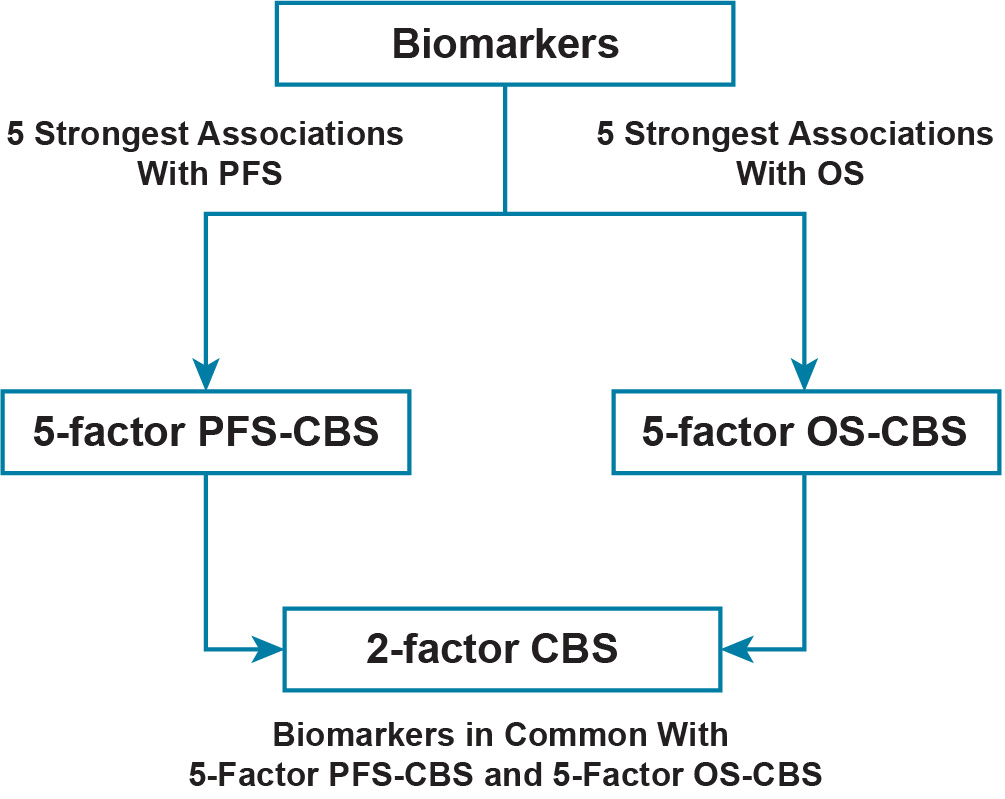


CBS, composite biomarker score; OS, overall survival; PFS, progression-free survival.

**Supplemental Figure 2.** (A) Median percent change in pharmacodynamic biomarker levels from baseline and (B) median percent change in select biomarker levels from baseline


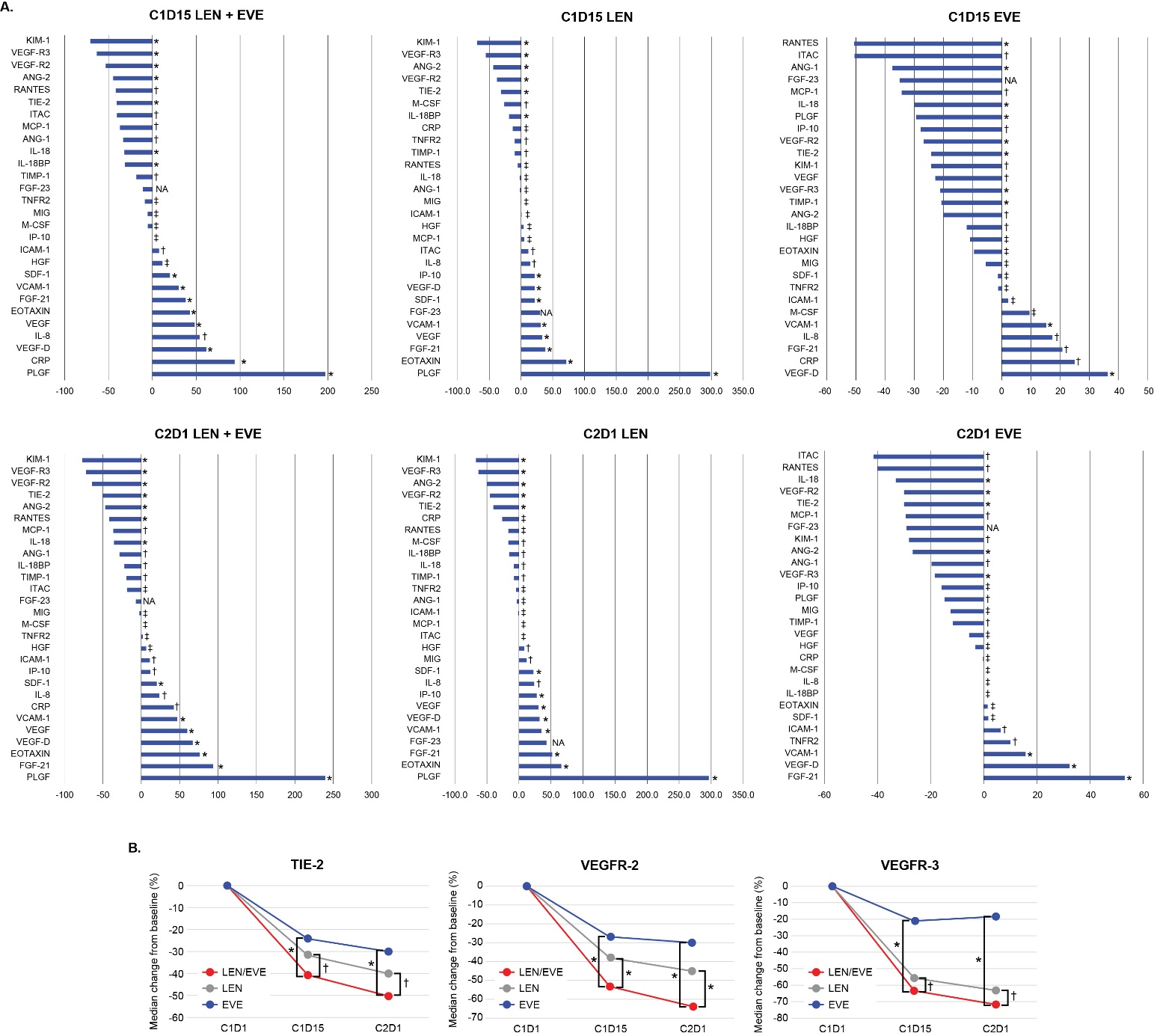


**P*<0.0001; †*P*<0.05; ‡*P*≥0.05

ANG-1, angiopoietin 1; ANG-2, angiopoietin 2; CRP, C-reactive protein; EOTAXIN1, also known as CCL11 or C-C motif chemokine ligand 11; EVE, everolimus; FGF-21, fibroblast growth factor 21; FGF-23, fibroblast growth factor 23; HGF, hepatocyte growth factor; ICAM-1, intercellular adhesion molecule 1; IL-18, interleukin 18; IL-18BP, interleukin 18-binding protein; IL-8, interleukin 8; IP-10, interferon gamma-induced protein 10; ITAC, interferon-inducible T-cell alpha [chemoattractant](https://en.wikipedia.org/wiki/Chemoattractant); KIM-1, kidney injury molecule 1; LEN, lenvatinib; M-CSF, macrophage colony-stimulating factor; MCP-1, monocyte chemoattractant protein-1; MIG, monokine induced by gamma interferon; PLGF, placental growth factor; RANTES, also known as CCLF, chemokine C-C motif ligand 5; SDF-1, stromal cell-derived factor 1; TIE-2, angiopoietin-1 receptor; TIMP1, tissue inhibitor of metalloproteinase-1; TNFR2, tumor necrosis factor receptor 2; VCAM-1, vascular cell adhesion protein 1; VEGF, vascular endothelial growth factor; VEGF-D, vascular endothelial growth factor D; VEGFR-2, vascular endothelial growth factor receptor 2; VEGFR-3, vascular endothelial growth factor receptor 3.

**Supplemental Figure 3.** ORR in OS-CBS-high (5-factor) groups and OS-CBS-low groups

**
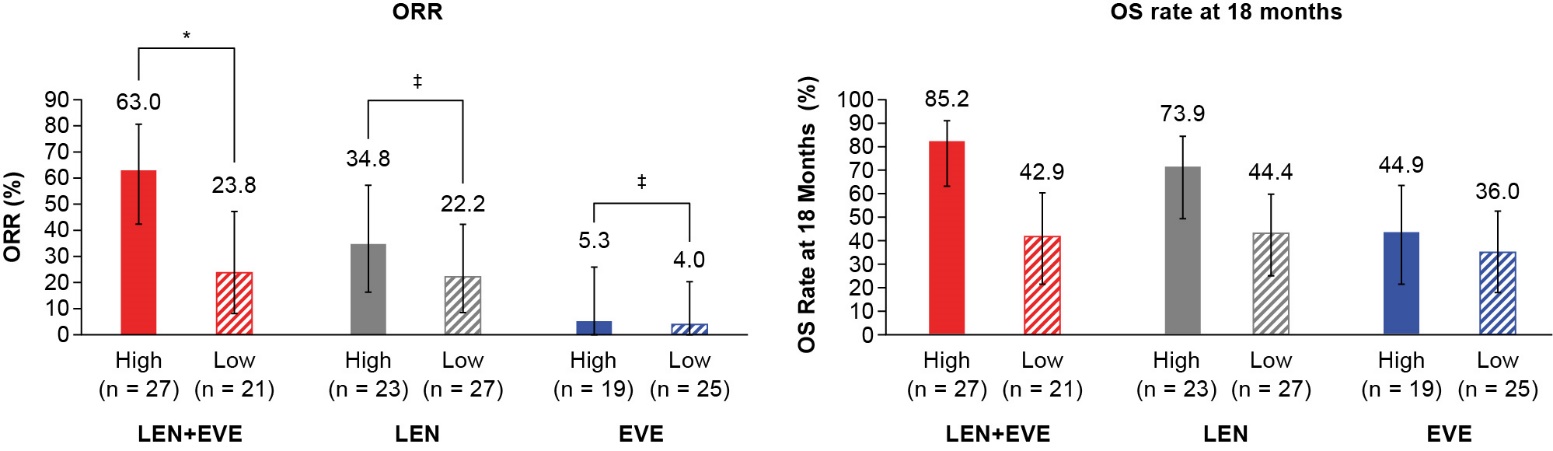
**

**P*<0.01; †*P*<0.1; ‡*P*≥0.1.

CBS, composite biomarker score; EVE, everolimus; LEN, lenvatinib; OS, overall survival; ORR, objective response rate.

**Supplemental Figure 4.** Kaplan–Meier curves of OS for CBS 2-factor CBS-high groups compared with CBS-low groups within treatment arms. (A) lenvatinib + everolimus, (B) lenvatinib, and (C) everolimus


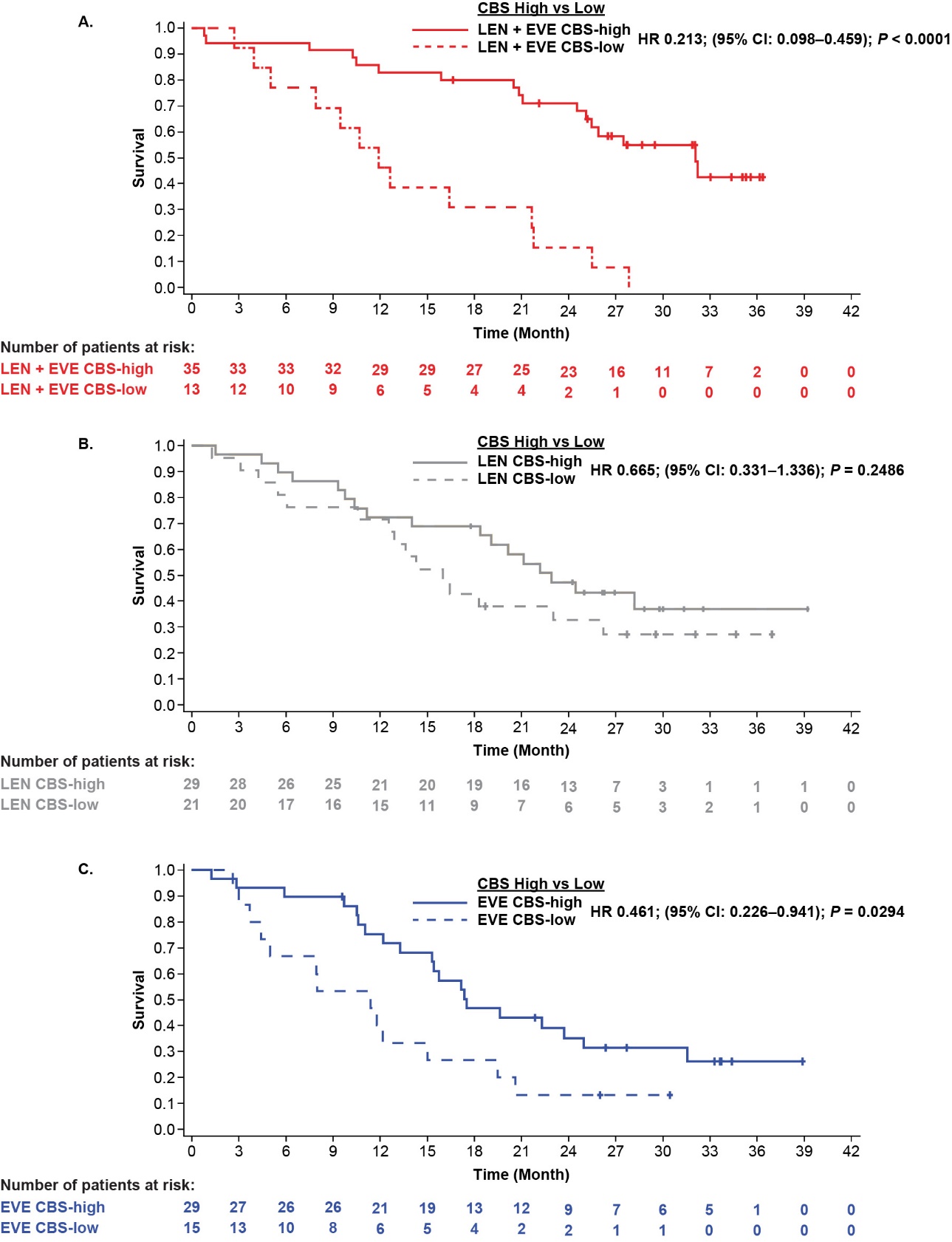


CBS, composite biomarker score; CI, confidence interval; EVE, everolimus; LEN, lenvatinib; NE, not estimable; OS, overall survival.
